# Geographical Distribution Dynamics of *Acorus calamus* in China Under Climate Change

**DOI:** 10.3390/plants13233352

**Published:** 2024-11-29

**Authors:** Chunlei Yue, Hepeng Li, Xiaodeng Shi

**Affiliations:** Zhejiang Academy of Forestry, Hangzhou 310023, China

**Keywords:** *Acorus calamus*, MaxEnt, ENMeval package, land use, human

## Abstract

*Acorus calamus*, a perennial emergent herb, is highly valued for its ornamental appeal, water purification ability, and medicinal properties. However, there is a significant contradiction between the rapidly increasing demand for *A. calamus* and the diminishing wild resources. Understanding its geographical distribution and the influence of global climate change on its geographical distribution is imperative for establishing a theoretical framework for the conservation of natural resources and the expansion of its cultivation. In this study, 266 distribution records of *A. calamus* and 18 selected key environmental factors were utilized to construct an optimal MaxEnt model via the ENMeval package. We simulated the potential geographical distributions under current conditions and under three different climate scenarios (SSP126, SSP370, and SSP585) in the 2050s, 2070s, and 2090s. Additionally, we employed the jackknife method and response curves to identify the environmental factors with the greatest influence on the distribution of *A. calamus*, and their response intervals. The results indicate that the regularization multiplier (RM) of 3.5 and the feature combinations (FC) of linear (L), quadratic (Q), hinge (H), and product (P) are the optimal model parameter combinations. With these parameters, the model predictions are highly accurate, and the consistency of the results is significant. The dominant environmental factors and their thresholds affecting the distribution of *A. calamus* are the precipitation of the wettest month (≥109.87 mm), human footprint (≥5.39), annual precipitation (≥388.56 mm), and mean diurnal range (≤12.83 °C). The primary land use types include rivers and channels, reservoirs and ponds, lakes, urban areas, marshes, other constructed lands, rice fields, forested areas, and shrublands. Under current climate conditions, the suitable geographical distribution of *A. calamus* in China is clearly located east of the 400 mm precipitation line, with high- and low-suitability areas covering 121.12 × 10^4^ km^2^, and 164.20 × 10^4^ km^2^, respectively. Under future climate conditions, both high- and low- suitability areas are projected to increase significantly, whereas unsuitable areas are expected to decrease, with the centroid of each suitability zone shifting northward. This study provides a theoretical foundation for sustainable utilization, future production planning, and the development of conservation strategies for wild germplasm resources of *A. calamus*.

## 1. Introduction

The geographical distribution patterns of plant populations are predominantly influenced by climate, which plays a critical role in shaping plant growth and survival [1]. Global warming has now become a widely accepted international consensus [2]. According to recent studies, the average global surface temperature increase may have already exceeded 1.5 °C and could surpass 2 °C by the end of this decade [3]. The impact of global climate change is becoming increasingly pronounced. The increase in climate anomalies as a result of global warming has resulted in the increased occurrence and severity of extreme weather events, notably high temperatures and droughts, which are becoming more prominent each year [4]. This situation has severely impacted the global ecological environment and significantly disrupted the structure and function of terrestrial ecosystems, community composition, and species distribution patterns [5]. Global climate change may lead to shifts and alterations in the distribution patterns of most species, habitat loss, and fragmentation, and the extinction of endangered species with narrow natural distribution ranges [6]. Therefore, understanding the impacts of global climate change on the potential geographical distribution of plants is vital for assessing their responses to climate variations [7]. It is vital to evaluate the vulnerability of species and develop appropriate adaptive management strategies under changing climate conditions [8]. This knowledge is highly important for the conservation, utilization, and sustainable development of plant species.

Species distribution models (SDMs) have demonstrated an excellent ability to predict changes in the potential distribution areas of various species under global climate change [9]. These methods are widely applied in climate change research, ecosystem functional community analysis, plant diversity conservation, and biological invasion assessment, among other fields [10]. SDMs can be used to systematically evaluate the probability of species suitability in different regions by correlating species distribution information with corresponding environmental variables [11]. In theory, species distribution models can predict the distribution of species on a global scale and are expected to be used to guide species introduction efforts to suitable areas. Commonly used SDMs include the Maximum Entropy Model (MaxEnt), Genetic Algorithm for Rule-Set Production (GARP), Bioclim Domain, Climex, and Ecological Niche Factor Analysis (ENFA), among others [12]. Different models require varying data and algorithms for species distribution simulations, leading to potential discrepancies in the calculated results [6]. Among these, the MaxEnt model has become one of the most widely used SDMs due to its stable simulation results and high accuracy [13]. This model can avoid overfitting and maintain the stability and reliability of prediction accuracy even under conditions of incomplete data or small sample sizes [14]. Additionally, this model can directly generate spatial maps of potentially suitable habitats and assess the contributions of environmental variables through jackknife testing [15]. It has been widely used in various research fields to simulate the potential distribution areas of species and evaluate their responses to climate change, biodiversity conservation, and biological evolution [16].

*Acorus* is a unique genus within monocotyledons and belongs to the family Acoraceae [17]. It was previously classified under the Araceae family, but modern taxonomic studies indicate that it should form its own family and be sister to all other monocots [18]. It has garnered significant attention due to its traditional medicinal value. According to the widely accepted classifications by the Flora of China (FOC), the Plant List, and the International Plant Names Index (IPNI), only two species within the *Acorus* genus are recognized globally: *Acorus calamus* and *Acorus gramineus*. However, several studies have shown that this genus contains many more species that have been presumed before, especially in Eurasia [17,18]. *Acorus calamus* contains one species (*A. calamus* L. var. *calamus*) and one variety (*A. calamus* L. var. *verus* L.), both of which belong to the genus *Acorus* of Acoraceae and are important perennial emergent herbs; this study does not distinguish between them [19]. It is widely distributed from the northern temperate zone to subtropical regions, and China serves as the primary distribution center for the *Acorus* genus [20].

*A. calamus* has an attractive appearance, with most of its stems and leaves standing upright above the water surface, while the lower stems and roots grow underwater. It has high ornamental value and is often used as a feature in water gardens or as a plant in constructed wetlands [21]. Recent studies have shown that, in addition to its great ornamental value, *A. calamus* has significant applications in water remediation due to its excellent water purification capabilities; thus, it is commonly used to remediate polluted water bodies, such as domestic sewage [22], and heavy-metal-contaminated water sources, including wastewater containing cadmium [23] and zinc [24].

Moreover, *A. calamus* is known for its aromatic properties, with its rhizomes being used in traditional medicine [25]. The plant is characterized by its pungent, bitter, and warm nature, offering various pharmacological benefits, such as insect repellent, analgesic, antibacterial, antifungal, antianxiety, antioxidant, and renoprotective effects [26]. It holds significant potential for development and application in the healthcare sector [27]. The essential oil extracted from *A. calamus* contains monoterpenes, sesquiterpenes, alkaloids, anthraquinones, aromatic essential oils, and flavonoids [28], making it an excellent ingredient for food flavoring and cosmetic fragrances. In many Asian countries, *A. calamus* is used as a substitute for other spices, such as ginger and cinnamon [29]. In recent years, the demand for products derived from *A. calamus* has increased due to the continuous development of related medicinal formulations [30].

Although *A. calamus* is widely distributed, natural populations are often confined to small areas along riverbanks, streams, or marshy wetlands [31]. The natural seedling rate is extremely low, and rhizome branches grow slowly, making them vulnerable to extensive root harvesting [20]. As a result, the natural resources of *A. calamus* are highly susceptible to damage. More critically, the current exploitation of *A. calamus* heavily depends on wild germplasm resources. With the increasing demand for *A. calamus*, wild populations are being subjected to unrestrained harvesting, leading to a sharp decline in resource reserves [32]. Currently, research on *A. calamus* has mainly focused on areas such as water pollution control, endophytic fungal inhibition, heavy metal accumulation, and water stress [33]. However, there are few reports on the suitable geographical distribution areas for *A. calamus*, despite its importance as a key ornamental and medicinal plant.

Therefore, there is an urgent need to study the suitable geographical distribution of *A. calamus* to understand its precise growth range and expand artificial cultivation areas to meet market demand. Additionally, considering the substantial pace of global climate change, it is crucial to examine how these changes affect its geographical distribution. This undertaking will facilitate the development of effective strategies for *A. calamus* introduction, cultivation, resource conservation, and adaptation to future global climate change. Moreover, this study offers a theoretical foundation for the sustainable use of *A. calamus*, aiding in future production planning and the development of strategies for protecting wild germplasm resources.

Based on the characteristics of China’s climate under global climate change, temperature and precipitation are expected to increase to varying degrees in the northeastern and southwestern regions of China [34]. We hypothesize that the future suitable geographical distribution of *A. calamus* will expand but become more fragmented. To verify our hypothesis, we utilized an optimized MaxEnt model based on the ENMeval 0.3.0 package to focus on the following objectives: (1) to investigate the potential geographic distribution of *A. calamus* in China under current climate conditions; (2) to identify the dominant environmental factors influencing the geographic distribution and determine the optimal conditions for *A. calamus*; and (3) to predict and compare the potential geographic distribution and migration trends under different climate scenarios in the 2050s (2041–2060), 2070s (2061–2080), and 2090s (2081–2100) against the backdrop of global warming.

## 2. Materials and Methods

### 2.1. Occurrence Data

**Figure 1:**
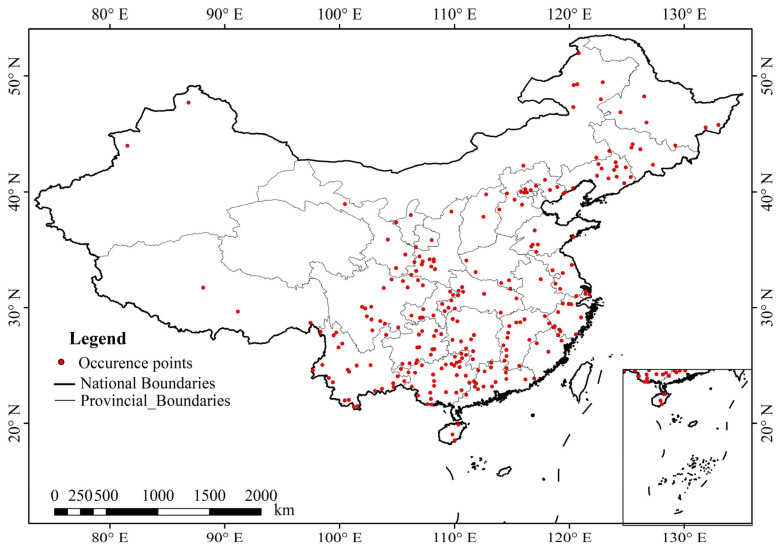
Distributions of occurrence points of *A. calamus*.

The study area was located in China (3.85–53.55° N, 73.55–135.08° E), where *A. calamus* commonly grows in wetland environments such as riverbanks, marshes, or lakeshores, typically at altitudes below 2600 m [31]. It thrives in warm, humid climates with abundant sunlight, with an optimal growth temperature range of 20–25 °C. Growth halts when temperatures fall below 10 °C [31]. During winter, its rhizomes retreat into the soil to overwinter. It prefers loose, well-aerated soils with good water retention and nutrient-holding capacity and can tolerate periods of waterlogging [31]. In shallow water environments, *A. calamus* grows well, with lush foliage and thick rhizomes; however, under flooded conditions, its growth slows or even halts [35]. In this study, the distribution data of *A. calamus* were sourced mainly from the Global Biodiversity Information Facility (GBIF, http://www.gbif.org/), the Chinese Virtual Herbarium (CVH, http://www.cvh.ac.cn/), relevant literature, and field surveys conducted from May 2022 to May 2023. A total of 339 distribution points of *A. calamus* were collected. Duplicate coordinates and points with unclear geographic coordinates were removed. Additionally, for points with distances less than 5 km between them, only one point was retained to reduce the potential clustering effects that could lead to model overfitting [36]. This process resulted in a final dataset of 266 distribution points of *A. calamus* for modeling (Figure 1).

### 2.2. Filtering and Data Processing of Environmental Variables

In this study, the selected environmental variables related to the geographical distribution of *A. calamus* included climatic, topographic, soil, human, and land variables (as listed in Table S1). The 19 climatic variables and elevation data were obtained from the WorldClim database (www.worldclim.org). The slope and aspect factors were derived from the elevation data. The periods included the current (1970–2000) and three future scenarios (2050s (2041–2060), 2070s (2061–2080), and 2090s (2081–2100)). In this study, three different SSP scenarios were used: SSP1-2.6 (SSP126), SSP3-7.0 (SSP370), and SSP5–8.5 (SSP585). SSPs are reference pathways that describe plausible scenarios in the evolution of society and ecosystems [37]. For future climate scenarios, we utilized the BCC-CSM2-MR climate change model from the Coupled Model Intercomparison Project Phase 6 (CMIP6) dataset [38].

Soil factor data were obtained from the Harmonized World Soil Database (HWSD) (http://webarchive.iiasa.ac.at/Research/LUC/External-World-soil-database/HTML/index.html?sb=1, accessed on 10 December 2023).

Human footprint data were collected from the International Geoscience InformationNetwork Center (https://sedac.ciesin.columbia.edu, accessed on 5 August 2024).

The land use dataset used was provided by the National Tibetan Plateau/Third Pole Environment Data Center (http://data.tpdc.ac.cn, accessed on 21 August 2024).

To avoid multicollinearity among the environmental variables leading to model overfitting, pre-modeling experiments were initially conducted using MaxEnt 3.4.1 with existing distribution data for *A. calamus* and 35 environmental variables. The objective of the experiment was to assess the predictive contributions of individual environmental factors to the geographical distribution of the species. SPSS 25.0 software (IBM Corp. Released 2017. IBM SPSS Statistics for Windows, Version 25.0. Armonk, NY, USA: IBM Corp.) was used to analyze the correlations among the environmental factors. Finally, considering both the contribution rates and correlation coefficient matrices, environmental factors with low contribution rates and correlation coefficients |r| > 0.8 were removed. Ultimately, 18 environmental factors with significant impacts on the potential geographical distribution of *A. calamus* were identified, as shown in Table S2.

The spatial resolution of all the ecological factor data is 2.5′. The administrative zoning map and map of China are from the National Geomatics Center of China (http://www.ngcc.cn/, accessed on 1 December 2023).

### 2.3. Model Optimization

The MaxEnt model, which is a sophisticated machine learning algorithm, is sensitive to sampling bias and susceptible to overfitting [39]. A review and assessment of recent MaxEnt-related literature revealed that using the default parameters of the model for data simulation may lead to inaccurate data analysis results and difficulties in evaluating the distributions of corresponding species [40]. The regularization multiplier (RM) and feature combination (FC) are important factors affecting the accuracy of MaxEnt model construction. In this study, the ENMeval 0.3.0 package [41] in R 3.6.1 software was utilized to adjust the RM and FC parameters. The RM was set from 0.5 to 4.0 with 8 levels, with adjacent levels spaced by 0.5. Six types of FCs were tested, namely, L, LQ, H, LQH, LQHP, and LQHPT, where L is linear, Q is quadratic, H is hinge, P is product, and T is threshold. The model performance was evaluated based on various criteria, including delta.AICc, avg.test.AUC (average test Area Under the Curve), the difference between the training and testing AUC values (AUC.DIFF), and a 10% omission rate (OR10) [42].

### 2.4. Model Construction

Upon importing the collected data points of the *A. calamus* distribution and the chosen environmental factors into the MaxEnt 3.4.1 modeling software, 25% of the data points were reserved as a test dataset to validate the model. The system convergence threshold was established at 10^−5^, and the maximum number of iterations was capped at 1000. The number of bootstrap replicates was set to 10. Random seeds were selected. Response curves and jackknife functions were chosen to analyze the impact of environmental factors on the *A. calamus* distribution. The RM and FC were set to the optimized parameters, while the rest were kept at the default settings.

### 2.5. Model Accuracy Evaluation

The model’s accuracy was evaluated using the area under the receiver operating characteristic (ROC) curve (AUC) and Cohen’s kappa values [39]. The AUC ranges from 0 to 1, with a higher AUC indicating greater model accuracy [43]. The kappa coefficient ranges from −1 to 1, where −1 signifies complete disagreement between the model predictions and sample data, and 1 signifies perfect agreement. Higher kappa values indicate better consistency and more reliable model predictions [44].

### 2.6. Geographical Distribution Classification

The suitability of a species’ geographical distribution is commonly expressed on a scale from 0 to 1, where higher values denote greater suitability for the species’ survival in the specified area [45]. In our study, the ASCII files produced by MaxEnt were imported into ArcGIS and transformed into raster files (TIFF format). With the “Reclassify” function, the potentially suitable geographical distribution areas for the species were classified into three levels: unsuitable (*p* < 0.2), low suitability (0.2 ≤ *p* < 0.4), and high suitability (*p* ≥ 0.4)

## 3. Results

### 3.1. Model Optimization and Accuracy Evaluation

In this study, we utilized the mature ENMeval package to optimize the parameters of the MaxEnt model to simulate the geographical distribution of *A. calamus*. The optimization results are shown in Table 1. The default settings included: feature combinations of linear (L), quadratic (Q), hinge (H), product (P), and threshold (T), with a regularization multiplier of 1. Under these settings, the average diff.AUC was 0.0422, the delta AICc was 130.7619, the avg.OR10 was 0.1884, and the avg.test.AUC was 0.8444. After optimization using the ENMeval package, the MaxEnt model settings were adjusted to feature combinations of linear (L), quadratic (Q), hinge (H), and product (P), with a regularization multiplier of 3.5. This optimization resulted in decreases in the avg.diff.AUC and avg.OR10 of 45.26% and 23.25%, respectively, and a delta AICc of 0, whereas the avg.test.AUC significantly increased compared with that before optimization. This finding indicates that the degree of overfitting in the model decreased by 45.26%, the complexity improved markedly, and the model’s transferability was enhanced. With the optimal parameters, the MaxEnt model was run 10 times, achieving an average training AUC of 0.8958 and an average testing AUC of 0.8849, both above 0.8 and higher than the random test AUC value (0.5). The kappa coefficient was 0.7733, demonstrating that the model’s predictive results were accurate and consistent. These results validate the high performance of the optimized MaxEnt model in accurately predicting the geographical distribution of *A. calamus*.

### 3.2. Environmental Factor Analysis

Through the optimized MaxEnt model simulation, the percent contribution and permutation importance values reveal that the six most significant environmental factors affecting the distribution of *A. calamus* are the precipitation of the wettest month (bio13), human footprint (humanfoot), land use (lucc), annual precipitation (bio12), mean diurnal range (bio2), and elevation. The cumulative values of the percent contribution and permutation importance for these factors are 80.47% and 84.87%, respectively (Table S3). According to the jackknife test results (Figure S1), the factors that significantly increase the regularized training gain of the model are humanfoot, bio13, and bio12. In summary, the distribution of *A. calamus* is influenced primarily by temperature and precipitation factors, including bio13, humanfoot, lucc, bio12, bio2, and elevation. Soil factors have minimal impacts on the current distribution of *A. calamus* (Table S3 and Figure S1).

**Figure 2:**
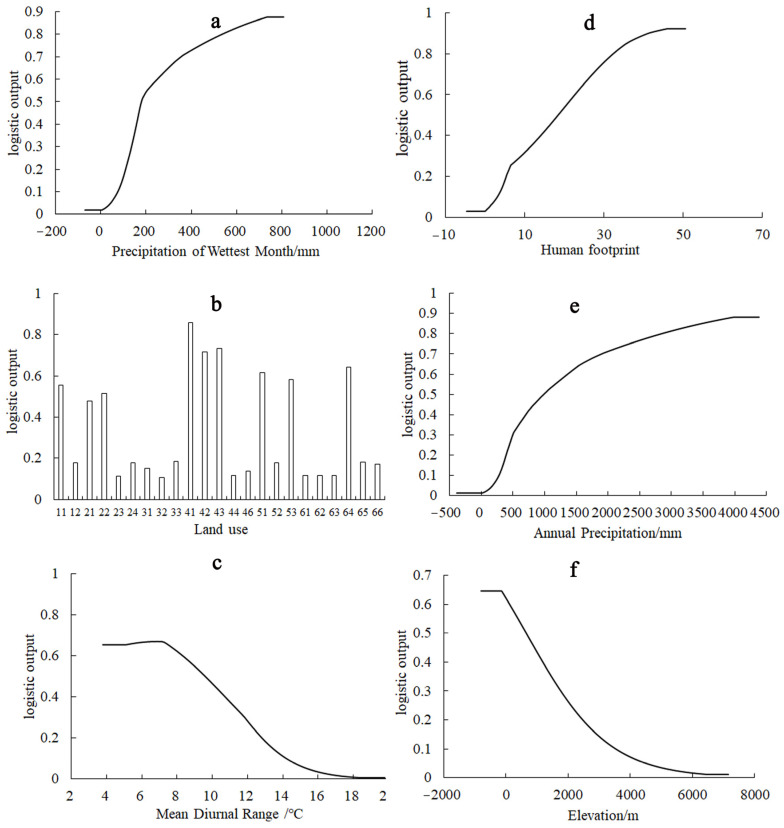
Response curves of the six major environmental factors. (**a**) Precipitation of the wettest month; (**b**) Land use; (**c**) Mean diurnal range; (**d**) Human footprint; (**e**) Annual precipitation; (**f**) Elevation.

An examination of the response curves of *A. calamus* to the six dominant environmental factors revealed a trend where the probability of distribution initially increased and eventually stabilized as the concentrations of bio13, humanfoot, and bio12 increased (Figure 2). Conversely, an increase in bio2 and elevation led to a decrease in the distribution probability (Figure 2). The primary land use types for the distribution of *A. calamus* are rivers and channels, reservoirs and ponds, and lakes. When the distribution probability of *A. calamus* in a suitable geographical distribution exceeds the threshold (0.2), the dominant environmental factors that restrict its distribution fall within the following ranges: bio13 ≥ 109.87 mm, humanfoot ≥ 5.39, bio12 ≥ 391.25 mm, bio2 ≤ 12.83 °C, elevation ≤ 2469.24 m and land use types include rivers and channels, reservoirs and ponds, lakes, urban areas, marshes, other constructed lands, rice fields, forested areas, and shrublands, as shown in Figure 2.

### 3.3. Current Potential Geographical Distribution

**Figure 3:**
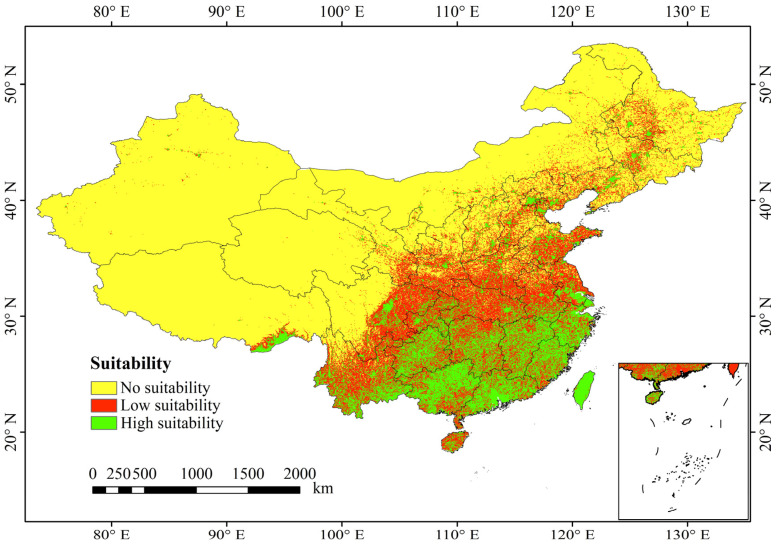
Potential geographical distribution pattern of *A. calamus* in China under current climatic conditions.

The results of the simulated current distribution of *A. calamus* via the optimized MaxEnt model are shown in Figure 3. In this figure, the green areas represent high-suitability distribution zones, the red areas represent low-suitability distribution zones, and the yellow areas represent unsuitable distribution zones (similarly indicated in subsequent figures). The figure shows that the suitable geographical distribution areas for *A. calamus* in China are distinctly located east of the 400 mm isohyet line. However, its distribution is highly fragmented. High-suitability distribution areas are found primarily in southeastern Tibet, eastern and southern Yunnan, Guizhou, Guangxi, Guangdong, Hunan, Jiangxi, Fujian, Zhejiang, and most of Taiwan. There are also scattered distributions in central Hainan, central Sichuan, southern Jiangsu, Henan, Shandong, Shanxi, Shaanxi, Beijing, Liaoning, Jilin, and Heilongjiang, covering 121.12 × 10^4^ km^2^, which accounts for 12.61% of the study area. The low-suitability distribution areas include most of Hainan, northern Yunnan, eastern Sichuan, northern Chongqing, southern Gansu, southern Shaanxi, southern Ningxia, Shanxi, Hebei, Hubei, Henan, Anhui, and Shandong. Eastern Inner Mongolia, northern Liaoning, Jilin, Heilongjiang, and southeastern Tibet (above the medium-suitability areas) also have notable distributions. These regions cover 164.20 × 10^4^ km^2^ accounting for 17.09% of the study area. Additionally, regions where *p* < 0.2 are considered unsuitable for the growth of *A. calamus*; these regions cover 675.27 × 10^4^ km^2^, which accounts for 70.30% of the area in China.

### 3.4. Future Geographical Distribution

**Figure 4:**
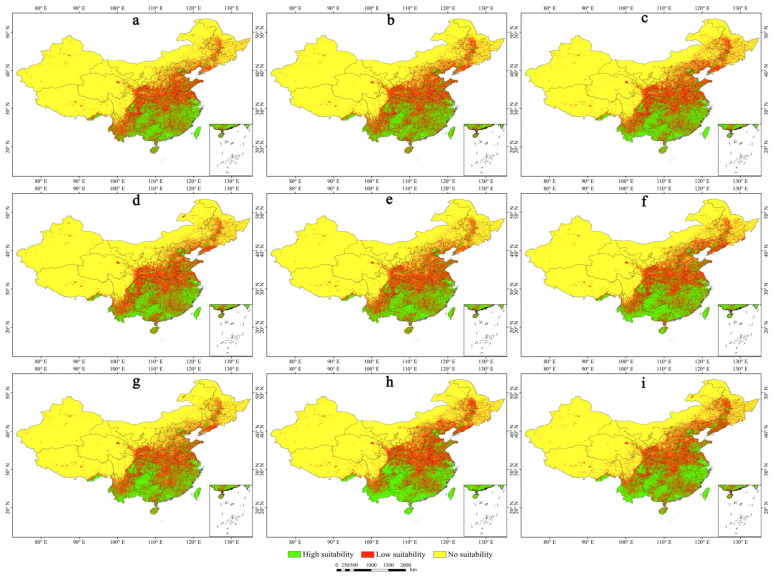
Future geographical distribution maps of *A. calamus.* (**a**) 2050s-SSP126; (**b**) 2050s-SSP370; (**c**) 2050s-SSP585; (**d**) 2070s-SSP126; (**e**) 2070s-SSP370; (**f**) 2070s-SSP585; (**g**) 2090s-SSP126; (**h**) 2090s-SSP370; (**i**) 2090s-SSP585.

**Figure 5:**
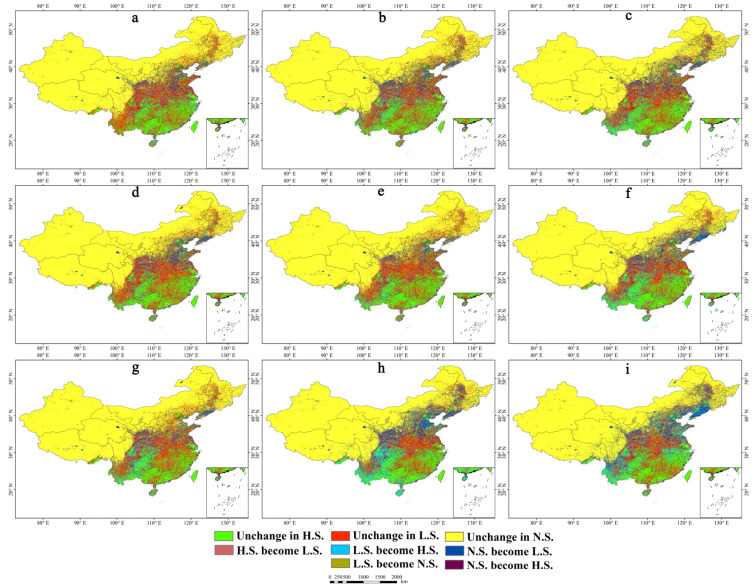
Future dynamic changes of different grade distribution areas. (**a**) 2050s-SSP126; (**b**) 2050s-SSP370; (**c**) 2050s-SSP585; (**d**) 2070s-SSP126; (**e**) 2070s-SSP370; (**f**) 2070s-SSP585; (**g**) 2090s-SSP126; (**h**) 2090s-SSP370; (**i**) 2090s-SSP585.

Following the same standards as in the previous sections, predictions were made for the distribution areas of *A. calamus* for three future periods under three target scenario pathways. The resulting future geographical distribution maps are shown in Figure 4. Dynamic change maps for each distribution grade are shown in Figure 5, and the areas and variation ranges of each suitability zone are presented in Table 2. The comprehensive results indicate that under the three shared socioeconomic pathways and representative concentration pathway climate scenarios for the 2050s, 2070s, and 2090s, the locations and areas of the *A. calamus* distribution at the different suitability levels will undergo varying degrees of change. Both the high-suitability and low-suitability areas are projected to significantly expand, whereas unsuitable areas are expected to decrease.

In the future, the area of high-suitability distribution zones for *A. calamus* will first increase and then decrease over time under the SSP126 and SSP585 scenarios (Table 2). However, under the SSP370 scenario, the area will expand continuously, showing an overall increasing trend with decreasing fragmentation. The growth rate will range from 12.07% to 40.46% (Table 2). Notably, under the SSP585 scenario in 2090, the high-suitability distribution area will reach its maximum of 170.13 × 10^4^ km^2^, representing an increase of 40.46% compared with that under the current climatic conditions (Table 2, Figure 4i and Figure 5i). This expansion will mainly result from the transformation of low-suitability zones in most of Yunnan, eastern Sichuan, northern Guizhou, southern Hubei and Anhui, and the eastern Shandong provinces (Figure 5i).

In the future, under the SSP126 scenario, the low-suitability distribution zones for *A. calamus* will initially increase and then decrease over time. Under the SSP370 and SSP585 scenarios, the increase will initially decrease and then increase, with an overall increase ranging from 0.90% to 14.13% (Table 2). Notably, under the SSP585 scenario in 2050, the low-suitability distribution area will reach its maximum at 187.39 × 10^4^ km^2^ (Table 2). Compared with the current climatic conditions, the increase in area will come from the conversion of primarily the unsuitable zones in Liaoning, central Jilin, northern Heilongjiang, eastern Inner Mongolia, northern Hebei, northern Shanxi, central Ningxia, and southern Gansu, as well as sporadic unsuitable regions in eastern Heilongjiang, eastern Qinghai, western Sichuan, and eastern Tibet (Figure 4c and Figure 5c). In the future, the unsuitable zones for *A. calamus* will show a continuous decreasing trend with increasing radiative forcing over time, with an overall reduction ranging from 3.89% to 10.15% (Table 2).

### 3.5. Centroid Changes in the A. calamus Distribution Pattern

**Figure 6:**
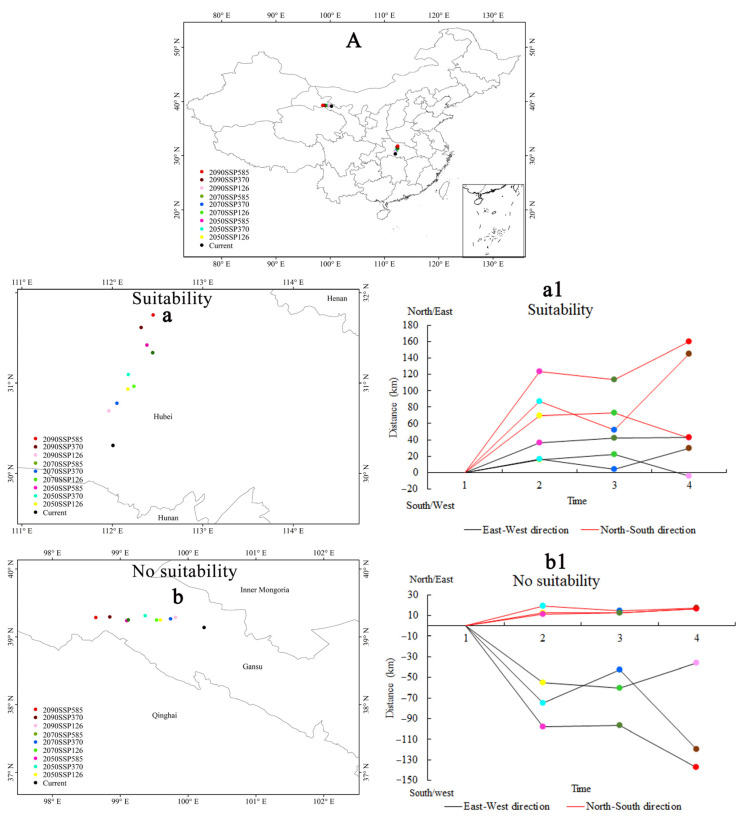
Future centroid movement of the potential geographical distribution area of *A. calamus* under different climate change scenarios. (**A**) Centroids in China; (**a**) Centroids in suitable area. (**b**) Centroids in unsuitable area; (**a1**,**b1**) indicates the distances that centroids of different grades of suitable distribution area migrate in two directions (north–south and east–west) under future climate change.

Figure 6 depicts the projected shifts in centroid positions within the potential geographical distribution areas of *A. calamus* across various climate change scenarios. Specifically, Figure 6(a1,b1) depict the movement distances of the distribution area centroids in the east-west, and north-south directions relative to the current centroid across the three periods and three climate change scenarios. As depicted in the figures, the centroid of the potential geographical distribution area for *A. calamus* is predominantly situated within Hubei Province, with a noticeable trend of northward migration across all future climate scenarios. In contrast, the centroids of unsuitable areas tend to move northwest.

## 4. Discussion

### 4.1. Model Accuracy

This study highlights the inaugural utilization of an optimized MaxEnt model to project the potential geographical distribution of the herbaceous plant *A. calamus* across China under present and future climate change scenarios. Among the various species distribution models, the MaxEnt model stands out for its robust predictive accuracy, even with limited sample sizes, narrow geographic ranges, and constrained environmental tolerances [46]. Consequently, it ranks among the most widely employed species distribution models [47]. Nevertheless, in practical applications of the MaxEnt model, optimizing model parameters is imperative to increase the precision and dependability of species distribution forecasts [48]. Compared with alternative data packages employed for optimizing the MaxEnt model, leveraging the ENMeval package to fine-tune the regularization multiplier and feature combination parameters yielded a model that demonstrated superior predictive performance [40]. The response curves were smoother, and the transferability of the model was greater, allowing for a more reasonable reflection of the response of a species to environmental factors and accurate simulations of potential species distribution. In this study, after the model parameters were optimized via the ENMeval package, the FC was set to LQPH, and the RM was set to 3.5. The optimized model achieved an AUC above 0.8 and a kappa coefficient above 0.7, with smooth and extended response curves. These findings indicate that the model constructed in this study has high predictive sensitivity and meets accuracy standards, resulting in highly accurate prediction outcomes. Relevant studies indicate that the optimal regularization multiplier is generally greater than the default value in MaxEnt software, with values typically between 2 and 4 yielding the best overall performance in predicting species distributions [49,50]. According to the results obtained from the MaxEnt model simulation in this study, the current distribution of *A. calamus* is distinctly located east of the 400 mm isohyet in China. This region almost entirely covers all the concentrated sample points and closely aligns with the dominant environmental factor of annual precipitation of ≥391.25 mm. This alignment further demonstrated that the model constructed in this study is highly precise and reliable. This finding also indicates that under current climatic conditions, the cultivation of *A. calamus* can be simply guided by selecting cultivation areas along the 400 mm isohyet.

### 4.2. Response of A. calamus to Dominant Environmental Factors

Climate and human factors are considered the most important driving factors controlling plant geographical distribution [51], and their influence on the geographical distribution of *A. calamus* is no exception. This study revealed that the dominant environmental factors influencing the distribution of *A. calamus* are the precipitation of the wettest month, the human footprint, land use, annual precipitation, the mean diurnal range, and elevation. Thus, the primary climatic factors affecting the geographical distribution of *A. calamus* are precipitation and temperature, which is consistent with the dominant environmental factors of the co-distributed wetland plant *Carex doniana* [14]. *A. calamus* is a typical wetland plant that has high water requirements. In this study, both of the top two dominant environmental factors influencing its distribution were related to precipitation. The rainfall during the wettest month reflects extreme moisture conditions, which typically occur in summer in China. Summer is the peak growing season for herbaceous plants, including the perennial *A. calamus* [45]. Additionally, annual precipitation indicates that *A. calamus* requires a substantial water supply for optimal growth. Summer is also the critical period for *A. calamus* to flower and fruit [51]. Adequate water during this time can increase atmospheric humidity and soil moisture, thereby promoting the growth of *A. calamus*.

Temperature plays an important role in regulating plant life activities and physiological processes, thereby shaping plant distribution [46]. Within certain thresholds, temperature can increase a plant’s adaptability to geographical distribution conditions and environmental fluctuations. However, exceeding a specific temperature threshold can cause the geographical distribution range of a plant to shrink or even disappear [52]. In this study, bio2 was ≤12.83 °C indicating that *A. calamus* thrives in regions with relatively small daily temperature fluctuations. Research has shown that *A. calamus* requires a certain amount of heat for growth, ceases to grow below 10 °C, and survives winter by submerging its rhizomes into the mud [27]. Elevation also constrains the potential geographic distribution of *A. calamus*. In the model, the elevation threshold for its potential distribution is 2469.24 m, which aligns with its natural distribution typically found below 2600 m [31], indicating *A. calamus*’ ability to adapt to a specific range of altitude variations.

Based on the aforementioned climate factors and thresholds, *A. calamus* thrives in warm and humid climates with abundant rainfall and minimal diurnal temperature variation. This finding aligns with those of previous studies [19]. *A. calamus* is predominantly found in the Yangtze River Basin and regions to the south, which exhibit significant seasonal changes but relatively mild temperature variations [27]. Consequently, the fundamental growth characteristics of *A. calamus* can be preliminarily analyzed based on the variations in these 6 key environmental factors across different regions.

The human footprint and land use have become the second and third most significant factors influencing *A. calamus* distribution, highlighting the substantial impact of human activities on its geographic range. Field surveys revealed that infrastructure projects such as waterway construction, road construction, farmland expansion, and urban development all disrupt *A. calamus* habitats, with some populations facing severe challenges such as habitat shrinkage and fragmentation (Figure 3). Among the primary land use types within the *A. calamus* distribution areas, urban and other constructed lands ranked the highest, further confirming that human activities are critical factors affecting and even constraining suitable *A. calamus* habitats. In this study, the model identified key land types in potential *A. calamus* distribution areas, including rivers, reservoirs, ponds, lakes, urban areas, marshes, other constructed lands, paddy fields, forested areas, and shrublands—many of which contain water sources. Naturally, Acorus species grow around wetlands such as mountain streams, ponds, and shallow lake areas [31], indicating distinctive habitat associations.

Therefore, some basic growth characteristics of *A. calamus* can be preliminarily analyzed based on the variations in six primary environmental factors across different regions in this study.

### 4.3. Changes in the Geographical Distribution Pattern of A. calamus

The results of this study suggest that the total geographical distribution area of *A. calamus* will markedly expand under various future climate scenarios, likely due to the sensitivity of this species to moisture. In the context of future climate warming and increased atmospheric moisture content, predictions indicate that precipitation in southern China will likely increase [53]. Additionally, the northward shift of the rain belt may lead to increased rainfall in previously drier northern regions [54], thereby expanding the geographical distribution of *A. calamus*. The largest increase in area is observed under the SSP58.5 climate scenario, suggesting that high-concentration greenhouse gas scenarios are favorable for the geographic expansion of *A. calamus*. Similar results have been reported in studies on other plants, such as *Prunus mume Siebold & Zucc.* [44], *Ageratina adenophora* R.M. King & H. Rob. [55], *Fritillaria cirrhosa* D. Don [56], and *Actinidia chinensis* Planch. [57], where the suitable geographical distribution areas were found to increase under scenarios with increased greenhouse gas emissions.

However, the changes in the suitable geographical distributions of different suitability levels for *A. calamus* under future climate scenarios vary. Both high-suitability and low-suitability distribution areas exhibit a northward expansion trend under future climate change. The most significant changes occur mainly in North China and Northeast China, where unsuitable areas transform into low-suitability areas, and low-suitability areas gradually convert into high-suitability areas. Notably, the high-suitability distribution area shows not only an increasing trend but also decreasing fragmentation. The fragmentation of plant habitats can directly impact plant reproduction, gene flow, and population connectivity [58]. Research suggests that future precipitation in northeastern China will show a significant increasing trend, with greater precipitation increases in high-emission scenarios than in low-emission scenarios and in drier areas than in wetter areas. These regions will meet the growth requirements of *A. calamus* under these scenarios, facilitating its growth and reproduction. This also accounts for the significant northward movement of the centroid of the suitable geographical distribution for *A. calamus* across all three projected climate scenarios. Recent studies have shown that most species in China are gradually migrating northward in response to global climate change [59].

In conclusion, the geographical distribution of *A. calamus*, a valuable plant resource, is expected to expand in China under the influence of global climate change. Policies and regulations should be established to protect and utilize this resource effectively, ensuring the continuity of its habitats. These results provide a foundation for formulating conservation strategies for *A. calamus* in response to global climate change.

## 5. Conclusions

This study is the first to utilize an optimized MaxEnt model to simulate the potential geographical distribution pattern of *A. calamus* in China under both current and future climate change scenarios. The dominant environmental factors affecting the geographical distribution of *A. calamus* in China are bio13, humanfoot, lucc, bio12, bio2, and elevation. Under current climate conditions, the suitable geographical distribution areas for *A. calamus* in China are located primarily east of the 400 mm isohyet. In future climate scenarios, both the high- and low-suitability distribution areas of *A. calamus* will significantly increase over time, whereas the unsuitable areas will decrease. The centroid of the potential geographical distribution area of *A. calamus* is projected to shift overall toward higher latitudes in the north. Our findings provide a theoretical basis for future efforts focused on the introduction, cultivation, and conservation of *A. calamus*, as well as for addressing the impacts of global climate change. We recommend large-scale artificial cultivation in regions such as Guangdong, Guangxi, and Fujian to meet market demand while mitigating the effects of human activities and climate change on its wild populations.

## Figures and Tables

**Table 1:** Predictive performance of the MaxEnt model with default and optimized parameter settings.

Settings	Regularization Multiplier	Feature Combination	Avg.Test.AUC	Avg.Diff.AUC	Avg.OR10	Delta.AICc
Default	1	LQHPT	0.8444	0.0422	0.1884	130.7619
Optimized	3.5	LQPH	0.8546	0.0231	0.1466	0

**Table 2:** Areas and variation ranges of the distribution area.

Scenario	Period	High Suitability Area		Low Suitability Area		Total Area(×10^4^/km^2^)	Total Change
		Area(×10^4^/km^2^)	Change	Area(×10^4^/km^2^)	Change		
current		121.12		164.20		675.25	
SSP126	2050s	137.93	13.87%	178.41	8.65%	644.23	−4.59%
	2070s	135.74	12.07%	181.63	10.62%	643.20	−4.75%
	2090s	144.21	19.06%	165.68	0.90%	650.68	−3.64%
SSP370	2050s	142.92	17.99%	184.65	12.46%	633.00	−6.26%
	2070s	139.46	15.14%	172.14	4.84%	648.97	−3.89%
	2090s	167.55	38.33%	180.90	10.17%	612.12	−9.35%
SSP585	2050s	147.86	22.07%	187.39	14.13%	625.32	−7.39%
	2070s	154.90	27.89%	177.58	8.15%	628.08	−6.98%
	2090s	170.13	40.46%	183.72	11.89%	606.72	−10.15%

## Data Availability

The data presented in this study are available as Supplementary Materials.

## Supplementary Materials

The following supporting information can be downloaded at: https://www.mdpi.com/article/10.3390/plants13233352/s1, Table S1: Environmental variables used in this study; Table S2: The environmental variables used in this study; Table S3: Percent contribution and permutation importance of each environmental variable of *A. calamus*; Figure S1: Jackknife test for the environmental variables.
