# Supplementary material for: Geographical Distribution Dynamics of Acorus calamus in China Under Climate Change
Source: Plants (Basel). 2024 Nov 29;13(23):3352. doi: 10.3390/plants13233352 (PMC11644658; doi:10.3390/plants13233352)
Supplement: Supplementary file 1 [file plants-13-03352-s001.zip › plants-3311531-Supplementary Materials.pdf]

Table S1. The environment variable

Type	Code	Description	Unit
Climate	bio1	Annual mean temperature	℃
	bio2	Mean diurnal range (Mean of monthly (max.temp.-min.temp.)	℃
	bio3	Isothermality (bio2 / bio7) (× 100)	
	bio4	Temperature seasonality (standard deviation*100)	
	bio5	Max temperature of the warmest month	℃
	bio6	Min temperature of the coldest month	℃
	bio7	Temperature annual range (bio5- bio6)	℃
	bio8	Mean temperature of the wettest quarter	℃
	bio9	Mean temperature of the driest quarter	℃
	bio10	Mean temperature of the warmest quarter	℃
	bio11	Mean temperature of the coldest quarter	℃
	bio12	Annual precipitation	mm
	bio13	Precipitation of the wettest month	mm
	bio14	Precipitation of the driest month	mm
	bio15	Precipitation seasonality (Coefficient of variation)	
	bio16	Precipitation of the wettest quarter	mm
	bio17	Precipitation of the driest quarter	mm
	bio18	Precipitation of the warmest quarter	mm
	bio19	Precipitation of coldest quarter	mm
Soil	t-pH	Topsoil pH (H_2O)	
	t-clay	Topsoil clay fraction	%
	t-bulk	Topsoil bulk density	kg/dm^3
	t-oc	Topsoil organic carbon	
	t-ece	Topsoil electric conductivity	
	t-esp	Topsoil Sodicity (ESP-exchangeable sodium percentage)	%
	t-gravel	Topsoil gravel content	%
	t-sand	Topsoil sand fraction	%
	t-silt	Topsoil silt fraction	%
	t-texture	Topsoil soil texture	
	t-usda	Topsoil USDA texture classification	
topographical	elevation	Elevation	m
	slope		°
	aspect		rad
Human	humanfoot	Human footprint	
Land	lucc	Land use	

Table S2. The environmental variables used in this study

Type	Code	Description	Unit
Climate	bio1	Annual mean temperature	℃
	bio2	Mean diurnal range (Mean of monthly (max.temp.-min.temp.)	℃
	bio6	Min temperature of the coldest month	℃
	bio10	Mean temperature of the warmest quarter	℃
	bio12	Annual precipitation	mm
	bio13	Precipitation of the wettest month	mm
	bio14	Precipitation of the driest month	mm
	bio15	Precipitation seasonality (Coefficient of variation)	
	bio17	Precipitation of the driest quarter	mm
Soil	t-pH	Topsoil pH (H_2O)	/
	t-clay	Topsoil clay fraction	%
	t-silt	Topsoil silt fraction	%
	t-oc	Topsoil organic carbon	/
Topographical	elevation	Elevation	m
	slope	/	°
	aspect	/	rad
Human	humanfoot	Human footprint	
Land	lucc	Land use	

Table S3. Percent contribution and permutation importance of each environmental variable of *A. calamus*

Code	Description	Percent contribution	Permutation importance
bio13	Precipitation of the wettest month	24.45	5.94
humanfoot	Human footprint	21.85	46.20
lucc	Land use	12.68	8.31
bio12	Annual precipitation	11.47	9.77
bio2	Mean diurnal range (Mean of monthly (max.temp.－min.temp.)	5.25	3.28
elevation	Elevation	4.76	11.38
bio10	Mean temperature of the warmest quarter	3.03	1.56
bio6	Min temperature of the coldest month	2.92	2.70
bio1	Annual mean temperature	2.42	0.46
t-pH	Topsoil pH (H_2O)	2.24	1.82
bio14	Precipitation of the driest month	1.74	0.62
aspect		1.59	2.93

slope		1.47	0.60
t-clay	Topsoil clay fraction	1.37	1.53
bio15	Precipitation seasonality (Coefficient of variation)	1.12	0.78
t-silt	Topsoil bulk density	0.97	1.49
bio17	Precipitation of the driest quarter	0.67	0.61
t-oc	Topsoil organic carbon	0.00	0.01

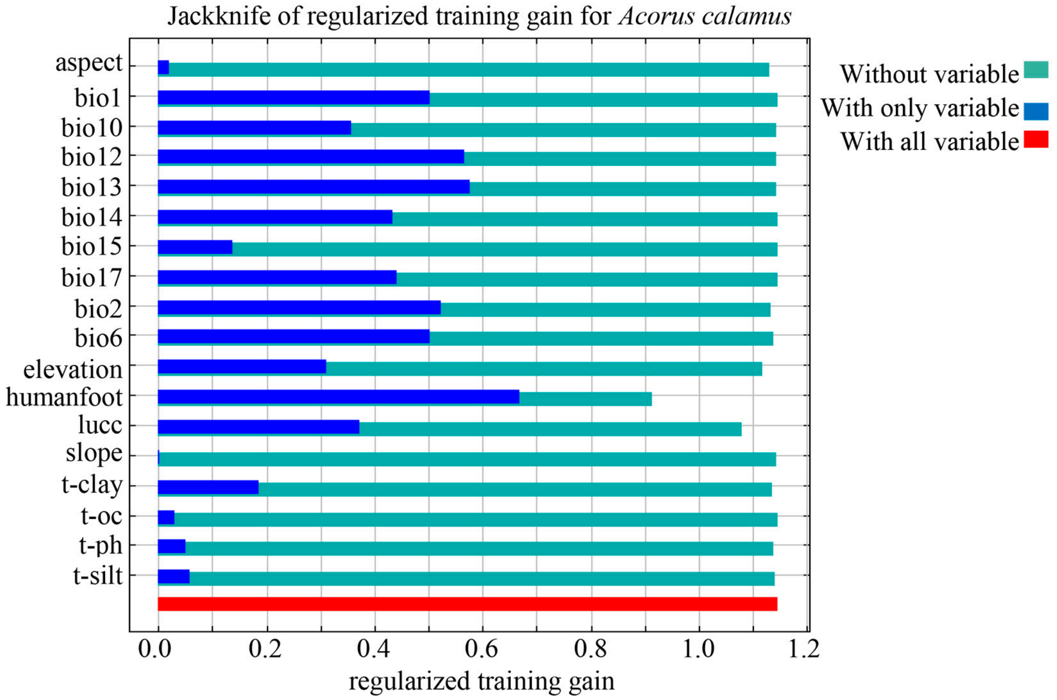

Fig. S1. Jackknife test for the environmental variables
